# Maternal outcomes of conservative management and cesarean hysterectomy for placenta accreta spectrum disorders: a systematic review and meta-analysis

**DOI:** 10.1186/s12884-024-06658-x

**Published:** 2024-07-05

**Authors:** Siman Pan, Minmin Han, Tianlang Zhai, Yufei Han, Yihan Lu, Shiyun Huang, Qing Zuo, Ziyan Jiang, Zhiping Ge

**Affiliations:** 1 School of Nursing, Nanjing Medical University, Nanjing, China; 2 Department of Obstetrics, Dongtai People’s Hospital, Affiliated Hospital of Nantong University, Yancheng, China; 3 Department of Obstetrics, Jiangsu Province Hospital, The First Affiliated Hospital of Nanjing Medical University, No.300, Guangzhou Avenue, Gulou District, Nanjing, Jiangsu 210029 China

**Keywords:** Placenta accreta, Maternal outcomes, Conservative management, Cesarean section, Hysterectomy, Placenta left in situ

## Abstract

**Background:**

Cesarean hysterectomy as a traditional therapeutic maneuver for placenta accreta spectrum (PAS) has been associated with serious morbidity, conservative management has been used in many institutions to treat women with PAS. This systematic review aims to compare maternal outcomes according to conservative management or cesarean hysterectomy in women with placenta accreta spectrum disorders.

**Methods:**

A systematic literature search was performed in MEDLINE, EMBASE, Cochrane Central Register of Controlled Trials, Web of Science, and four Chinese databases (Chinese Biomedical Literature Database, China National Knowledge Infrastructure, Chinese Wanfang database and VIP database) to May 2024. Included studies were to be retrospective or prospective in design and compare and report relevant maternal outcomes according to conservative management (the placenta left partially or totally in situ) or cesarean hysterectomy in women with PAS. A risk ratio (RR) with 95% confidence interval (95% CI) was calculated for categorical outcomes and weighted mean difference (WMD) with 95% CI for continuous outcomes. The Newcastle-Ottawa Quality Assessment Scale was used to assess the observational studies. All analyses were performed using STATA version 18.0.

**Results:**

Eight studies were included in the meta-analysis. Compared with cesarean hysterectomy, PAS women undergoing conservative management showed lower estimated blood loss [WMD − 1623.83; 95% CI: -2337.87, -909.79], required fewer units of packed red blood cells [WMD − 2.37; 95% CI: -3.70, -1.04] and units of fresh frozen plasma transfused [WMD − 0.40; 95% CI: -0.62, -0.19], needed a shorter mean operating time [WMD − 73.69; 95% CI: -90.52, -56.86], and presented decreased risks of bladder injury [RR 0.24; 95% CI: 0.11, 0.50], ICU admission [RR 0.24; 95% CI: 0.11, 0.52] and coagulopathy [RR 0.20; 95% CI: 0.06, 0.74], but increased risk for endometritis [RR 10.91; 95% CI: 1.36, 87.59] and readmission [RR 8.99; 95% CI: 4.00, 12.21]. The incidence of primary or delayed hysterectomy rate was 25% (95% CI: 19–32, *I*^*2*^ = 40.88%) and the use of uterine arterial embolization rate was 78% (95% CI: 65–87, *I*^*2*^ = 48.79%) in conservative management.

**Conclusion:**

Conservative management could be an effective alternative to cesarean hysterectomy when women with PAS desire to preserve the uterus and are informed about the limitations of conservative management.

**Prospero ID:**

CRD42023484578.

**Supplementary Information:**

The online version contains supplementary material available at 10.1186/s12884-024-06658-x.

## Introduction

Placenta Accreta Spectrum (PAS) disorders refer to the abnormal attachment of the placental trophoblast to the uterine myometrium resulting in partial or total retention of the placenta at the time of delivery [1–3]. Depending on the depth of placental implantation into the uterine myometrium, PAS disorders include placenta accreta, placenta increta, and placenta percreta [1]. PAS is one of the most life-threatening conditions during pregnancy, and can cause a series of serious maternal complications including severe postpartum hemorrhage (PPH), hemorrhagic shock, hysterectomy, multisystem organ failure, disseminated intravascular coagulation (DIC), and even death [2–5]. The incidence of PAS has been increasing in recent decades as cesarean delivery rates have increased worldwide, ranging from 1.7 to 4.6 per 10,000 deliveries according to a few prospective population-based studies [5–7].

The American College of Obstetricians and Gynecologists (ACOG) recommends cesarean hysterectomy as the principal and preferred treatment for women with PAS because of the high risk of excessive blood loss when removing the placenta [8]. However, cesarean hysterectomy as a traditional therapeutic maneuver causes secondary infertility and brings serious morbidity, especially in terms of massive blood loss and adjacent organ injury, becoming a major bothersome and troublesome disease of women of the reproductive period [9]. A study also showed that women with PAS disorders were more likely to report decreased quality of life, feelings of grief and depression after cesarean hysterectomy [3, 10]. Recently, conservative management (defined as the placenta left partially or totally in situ) has been widely advocated by experts as a means of preserving fertility and reducing rates of maternal morbidity [11]. One of the conservative managements presented by the International Federation of Gynecology and Obstetrics (FIGO) was to leave the placenta partially or totally in the uterine cavity allowing for its complete natural resorption [3, 12]. A recent retrospective multicenter study of 15 women with PAS managed with conservative managements showed that only 2 women underwent a delayed hysterectomy and the incidence of main complications was low [13]. Another retrospective cohort study also found that subsequent fertility was not affected in women following conservative management with PAS disorders [14].

A few retrospective or prospective observational studies that compared maternal outcomes according to conservative management and cesarean hysterectomy for women with PAS were presented. This study aims to summarize the findings of these published studies using meta-analytic methods in order to compare maternal outcomes such as estimated blood loss, units of packed red blood cells transfused, bladder injury, admission to intensive care unit (ICU), endometritis, primary or delayed hysterectomy and so on.

## Materials and methods

### Information sources and search strategy

The study was conducted in accordance with the Preferred Reporting Items for Systematic Reviews and Meta-Analyses (PRISMA) guideline 2020 [15]. We searched MEDLINE, EMBASE, Cochrane Central Register of Controlled Trials, Web of Science, and four Chinese databases (Chinese Biomedical Literature Database, China National Knowledge Infrastructure, Chinese Wanfang database and VIP database) to May 2024. The search strategy combined MeSH headings with free text words. There were no restrictions for language, date of publication, or geographic location. The details of the search strategy were shown in Table S1. The study protocol was registered with the International Prospective Register of Systematic Reviews (PROSPERO) (registration number: CRD42023484578).

### Eligibility criteria and study selection

All identified articles were transferred into EndNote X9. Literature selection was completed independently by two researchers according to inclusion and exclusion criteria, with inconsistencies were resolved by the third researcher. Firstly, duplicate literature was excluded, and only one copy was reserved. Furthermore, any literature irrelevant to the research topic was excluded by carefully screening the title and abstract. Finally, the literature included was determined by reading the full-text carefully.

#### Inclusion criteria

The participant was pregnant with PAS disorders and performed a cesarean delivery. In addition, eligible studies compared relevant maternal outcomes according to conservative management or cesarean hysterectomy for women with PAS. Furthermore, studies should be retrospective or prospective in design.

#### Exclusion criteria

The studies that did not compare maternal outcomes of conservative management and cesarean hysterectomy for PAS women were excluded. The studies that did not leave the placenta in situ in conservative management or did not involve cesarean delivery were excluded. Lastly, reviews, case reports and the studies published as conference abstracts were excluded.

### Data extraction and quality assessment

Data extraction was completed independently by two researchers and the following variables were recorded: author name, publication year, study location, study type, study time, sample size, patient characteristics and maternal outcomes. The primary outcome was estimated blood loss, and secondary outcomes included units of packed red blood cells transfused, bladder injury, endometritis and so on. Two independent researchers used the Newcastle-Ottawa Quality Assessment Scale (NOS Scale) to assess the studies. The NOS scale assessed literature quality from three components: selection of cohorts, comparability of cohorts, and outcomes in cohort studies [16]. According to the NOS scale score, the studies were classified as low quality literature (< 4 stars), medium quality literature (4–6 stars) and high-quality literature (7–9 stars).

### Data analyses

All analyses were performed using STATA version 18.0 (Stata Corporation, College Station, Texas, USA). The heterogeneity of studies was estimated using *I*^*2*^ test and *P*-values. If outcomes showed a significant heterogeneity (*I*^*2*^ ≥ 50%, *P* < 0.1), a random-effects model was used to analyze the data; if no significant heterogeneity (*I*^*2*^ < 50%, *P* ≥ 0.1) was shown, a fixed-effects model was used to analyze the data. *P*-value < 0.05 was considered statistically significant. The risk ratio (RR) with 95% confidence interval (95% CI) was calculated for categorical outcomes and weighted mean difference (WMD) with 95% CI was calculated for continuous outcomes. Due to a limitation in the number of studies and the judgment of funnel plot symmetry being relatively subjective [17], the method of quantitative detection of publication bias, including Egger’s test and Begg’s test were adopted. If outcomes showed publication bias (*P* < 0.05), the stability of the results was evaluated by Trim-and-fill method. Further subgroup analysis of outcomes with significant heterogeneity was conducted according to area income. And sensitivity analysis was performed.

## Results

### Study selection

**Fig. 1:**
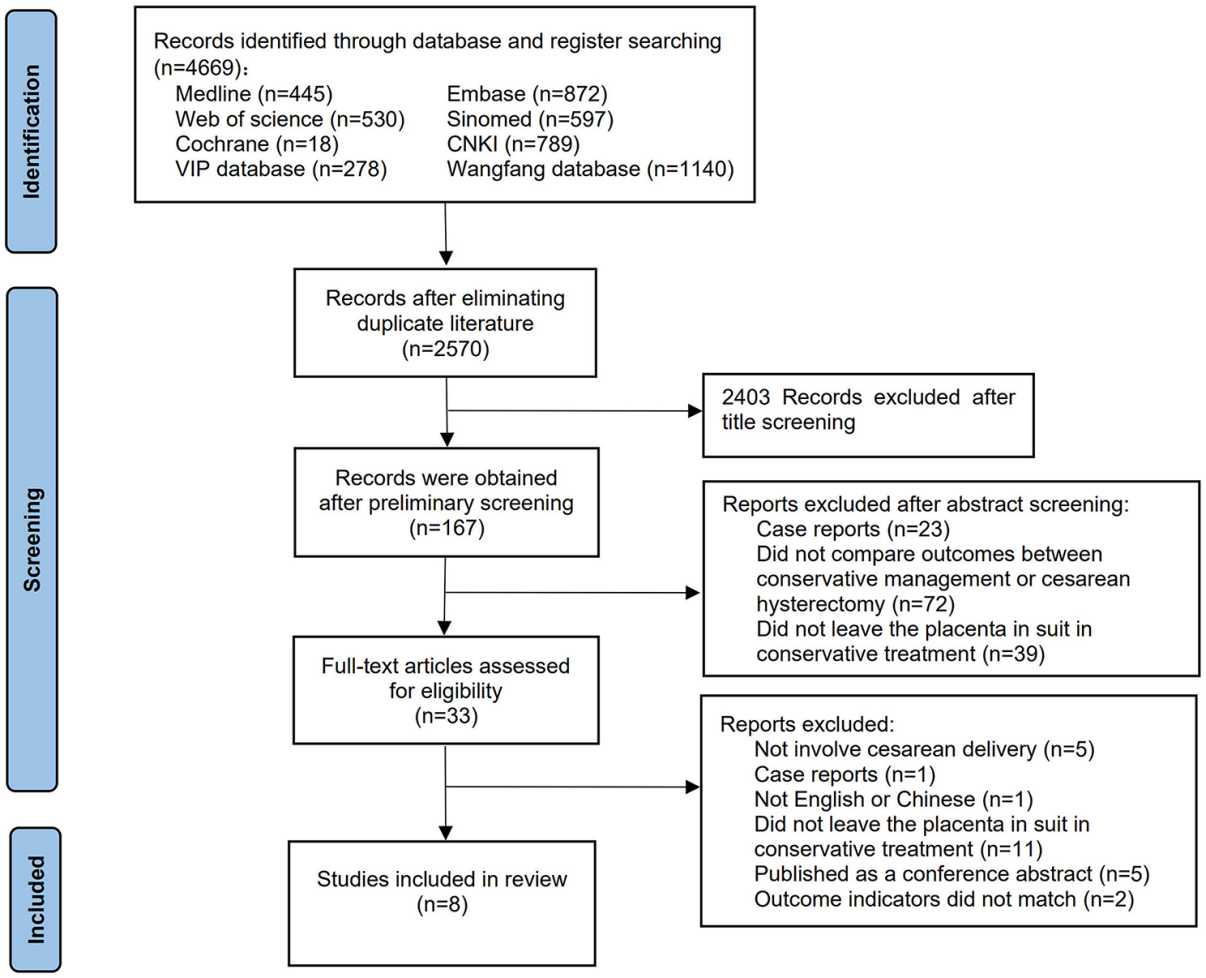
Study screening process

We identified 4669 studies through database and register searching, of which 2099 duplicates were automatically excluded. Moreover, 2403 studies were excluded from title screening and 134 studies from abstract screening. Ultimately, 8 studies were included in the review after full-text screening of 33 studies [5, 18–24]. The study selection process is shown in Fig. 1.

### Study characteristics

We included eight studies from eight countries and reported maternal outcomes of 579 women with placenta accreta after conservative management or cesarean hysterectomy. Moreover, 5 studies were retrospective in design and 3 studies were prospective in design. All but three were conducted in high-income areas. Detailed study characteristics were shown in Table 1.

**Table 1:** Summary of included studies

Reference	Country	Study design	Study time	Sample size	Participant characteristics	Maternal outcomes(Conservative vs. Hysterectomy)
Amsalem et al. [18]	Canada	Retrospective cohort study	2000–2009	26 (10 with conservative management and 16 with cesarean hysterectomy)	Women with placenta accreta and mean age of around 34.8 years; mean gravidity of 4.1; mean parity of 1.9; mean previous dilatation and curettage of 0.6; mean previous uterine scars of 1.8	Estimated blood loss (mL) (Mean, SD): 900 (754) vs. 3625 (2154); Packed red blood cells transfused (units) (Mean, SD): 2 (3.1) vs. 4.2 (2.3); Amount of fresh frozen plasma transfused (units) (Mean, SD): 0.7 (2.1) vs. 1.3 (1.6); Risk of bladder injury: RR 0.40 (95% CI: 0.05, 3.09); Risk of coagulopathy: RR 0.23 (95% CI: 0.03, 1.59); Hospital stay (days) (Mean, SD): 8.1 (3.4) vs. 11.8 (6.8); Risk of ICU admission: RR 0.53 (95% CI: 0.06,4.45)
Chung et al. [19]	Hong Kong, China	Retrospective cohort study	2005.5-2011.5	15 (6 with conservative management and 9 with cesarean hysterectomy)	Women with placenta accreta and mean age of around 36.5 years; mean gravidity of 4.5; mean parity of 1.5; mean previous cesarean section of 1.3; mean previous uterine curettage of 1.5	Estimated blood loss (mL) (Mean, SD): 627 (390) vs. 4544 (1506); Packed red blood cells transfused (units) (Mean, SD): 0.8 (1.2) vs. 9.8 (5); Risk of bladder injury: RR 0.48 (95% CI: 0.02,10.07); Risk of endometritis: RR 7.14 (95% CI: 0.40, 127.07); Risk of readmission: RR 15.71 (95% CI: 1.03, 240.75); Hospital stay (days) (Mean, SD): 18.1 (11.7) vs. 14.7 (12.4); Risk of ICU admission: RR 0.75 (95% CI: 0.09, 6.55); Mean operating time (min) (Mean, SD): 57 (18) vs. 148 (49)
Kutuk et al. [21]	Turkey	Retrospective cohort study	2010.5-2016.8	32 (15 with conservative management and 17 with cesarean hysterectomy)	Women with placenta accreta and mean age of around 33 years; mean gravidity of 4; mean parity of 2.5; mean previous cesarean deliveries of 1.5	Estimated blood loss (mL) (Mean, SD): 736 (647) vs. 2000 (751); Packed red blood cells transfused (units) (Mean, SD): 0.2 (0.4) vs. 0.9 (0.6); Amount of fresh frozen plasma transfused (units) (Mean, SD): 0.1 (0.2) vs. 0.5 (0.4); Risk of bladder injury: RR 0.25 (95% CI: 0.01,4.54); Hospital stay (days) (Mean, SD): 8.4 (5.2) vs. 6.8 (4.5); Mean operating time (min) (Mean, SD): 81 (13) vs. 146 (38)
El Gelany et al. [20]	Egypt	Retrospective cohort study	2017.1-2018.8	54 (16 with conservative management and 38 with cesarean hysterectomy)	Women with placenta accreta and mean age of around 32.5 years; 70% had more than three pregnancies; 78% had previous history of cesarean delivery; 35% had history of placenta previa	Estimated blood loss (mL) (Mean, SD): 2120 (870) vs. 2840 (1120); Packed red blood cells transfused (units) (Mean, SD): 2.9 (0.6) vs. 3.8 (1.2); Risk of bladder injury: RR 0.16 (95% CI: 0.02, 1.10); Risk of coagulopathy: RR 0.25 (95% CI: 0.01, 4.48); Risk of ICU admission: RR 0.30 (95% CI: 0.04, 2.18)
Lional et al. [22]	Singapore	Retrospective cohort study	2006.1-2017.12	74 (23 with conservative management and 51 with cesarean hysterectomy)	Women with placenta accreta and mean age of around 34 years; mean gravidity of 3; mean parity of 2; around 95.5% had previous history of cesarean delivery	Estimated blood loss (mL) (Mean, SD): 517 (2563) vs. 3169 (2563); Packed red blood cells transfused (units) (Mean, SD): 1.2 (5.8) vs. 7.2 (5.8); Risk of bladder injury: RR 0.07 (95% CI: 0.00, 1.20); Risk of readmission: RR 8.32 (95% CI: 3.10, 22.31); Mean operating time (min) (Mean, SD): 71 (98) vs. 172 (98)
Srinivasan et al. [23]	India	Retrospective and prospective observational study	2010–2020	34 (24 with conservative management and 10 with cesarean hysterectomy)	Women with placenta accreta and mean age of around 32.2 years; around 93% women were multigravida; around 82.5% had previous history of cesarean delivery; around 40% had history of dilatation and curettage	Estimated blood loss (mL) (Mean, SD): 1360 (563) vs. 2580 (737); Risk of bladder injury: RR 0.15 (95% CI: 0.01, 3.32); Risk of coagulopathy: RR 0.14 (95% CI: 0.02, 1.18); Risk of ICU admission: RR 0.09 (95% CI: 0.02, 0.35)
Sentilhes et al. [5]	France	Prospective observational study	2013.11-2015.10	148 (86 with conservative management and 62 with cesarean hysterectomy)	Women with placenta accreta and mean age of around 34.7 years; around 10.8% were nulliparous; around 79.7% had at least 1 previous uterine surgery; around 76.5% had history of placenta previa	Risk of bladder injury: RR 0.31 (95% CI: 0.08, 1.15); Risk of endometritis: RR 13.57 (95% CI: 0.81, 228.70); Risk of readmission: RR 8.531 (95% CI: 2.10, 34.71)
Paping et al. [24]	Germany	Prospective observational study	2020.1-2022.6	196 (10 with conservative management and 186 with cesarean hysterectomy)	Women with placenta accreta and mean age of around 34 years; mean gravidity of 3; mean parity of 2; mean previous cesarean deliveries of 2; around 85.7% had history of placenta previa	Estimated blood loss (mL) (Mean, SD): 1704 (614) vs. 2015 (295); Risk of bladder injury: RR 0.48(95% CI: 0.07, 3.13); Risk of ICU admission: RR 0.24 (95% CI: 0.04, 1.58)

*Abbreviations* RR, risk ratio; ICU, intensive care unit; CI, confidence interval; WMD, weighted mean difference; SD, standard deviation

### Risk of included studies bias

The results of the risk of bias assessment were shown in Table S2. All studies scored ≥ 7 on the Newcastle-Ottawa Quality Assessment Scale.

### Sensitivity analysis

Sensitivity analysis by the “leave-one-out” method found that our results were robust. In addition, for each maternal outcome, the original study results did not change substantially after each included study was excluded separately. The sensitivity analysis result of the primary outcome was shown in Figure S1.

### Synthesis of results

#### Maternal outcomes

**Fig. 2:**
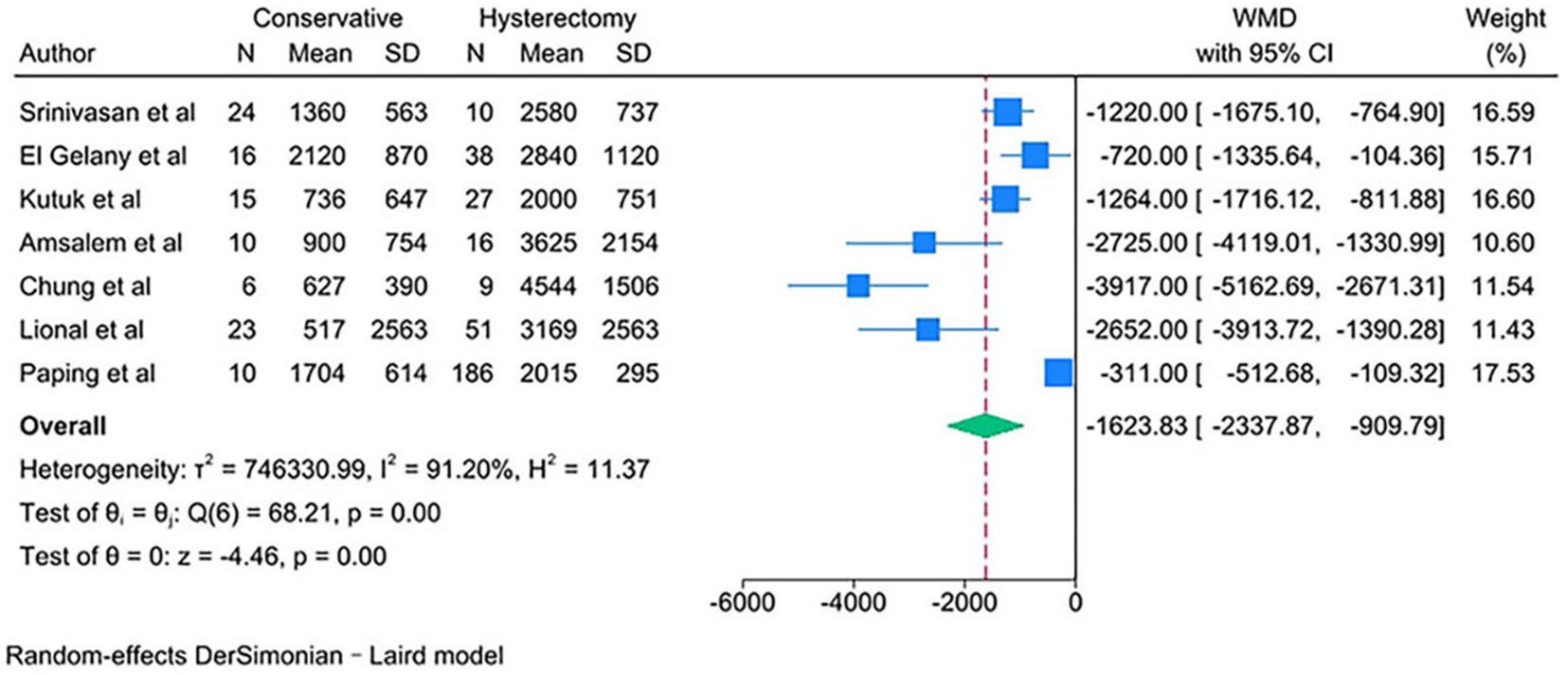
Estimated blood loss (mL) for PAS women undergoing conservative management or cesarean hysterectomy

**Fig. 3:**
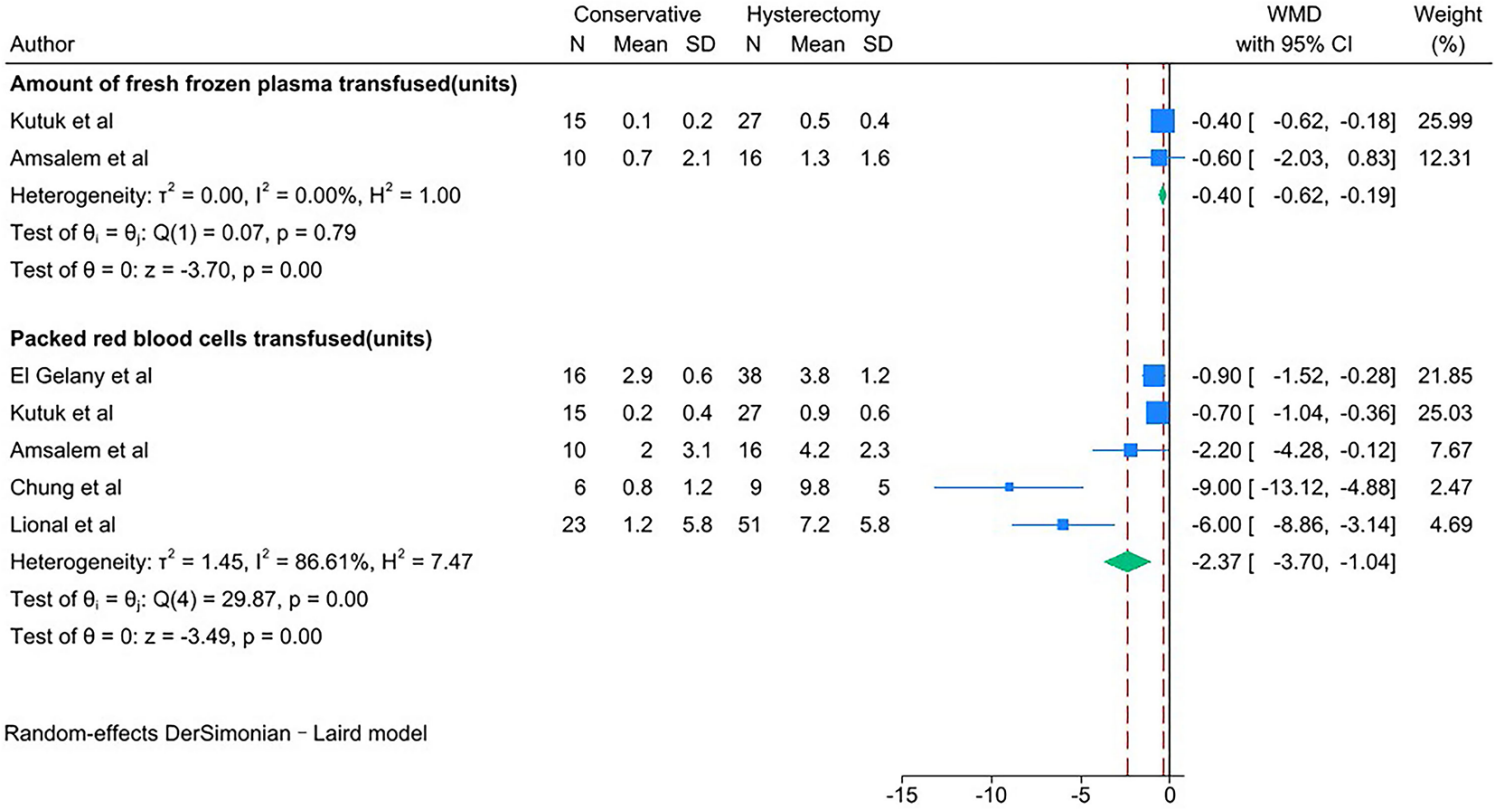
Units of blood transfused for PAS women undergoing conservative management or cesarean hysterectomy

PAS women with conservative management showed lower estimated blood loss (in mL) [WMD − 1623.83; 95% CI: -2337.87, -909.79; *I*^*2*^ = 91.20%] (Fig. 2), required fewer units of packed red blood cells transfused [WMD − 2.37; 95% CI: -3.70, -1.04; *I*^*2*^ = 86.61%] and units of fresh frozen plasma transfused [WMD − 0.40; 95% CI: -0.62, -0.19; *I*^*2*^ = 0.00%] (Fig. 3) than PAS women with cesarean hysterectomy.

**Fig. 4:**
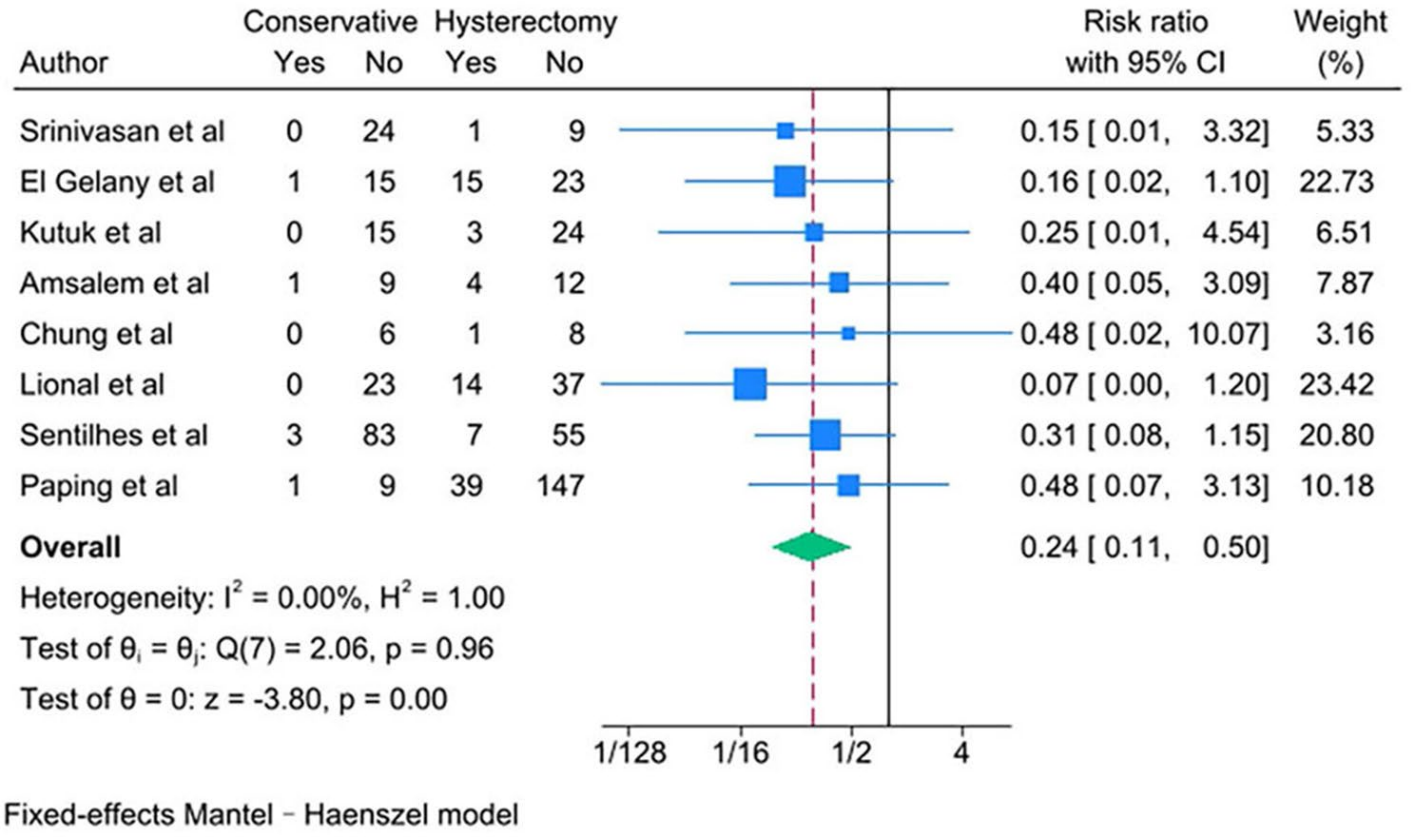
Bladder injury for PAS women undergoing conservative management or cesarean hysterectomy

**Fig. 5:**
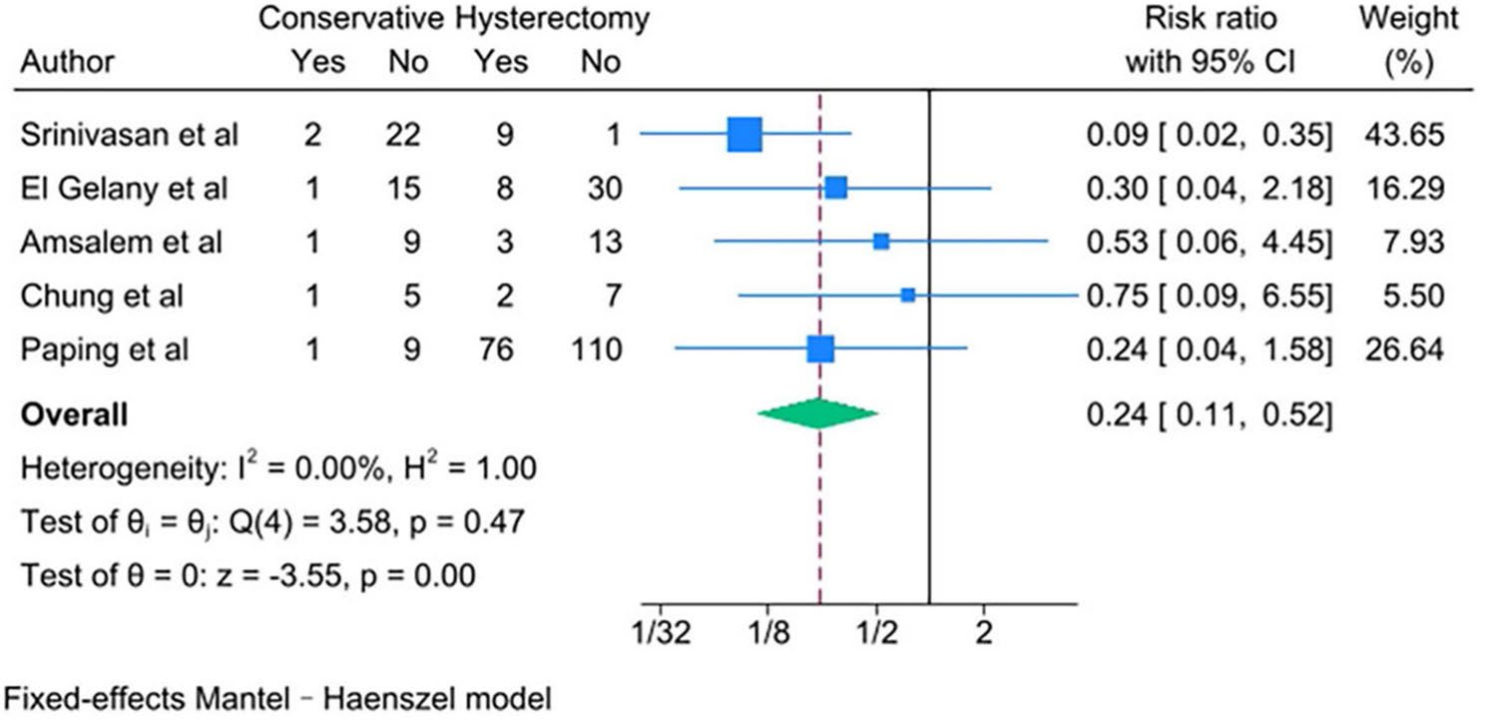
ICU admission for PAS women undergoing conservative management or cesarean hysterectomy

**Fig. 6:**
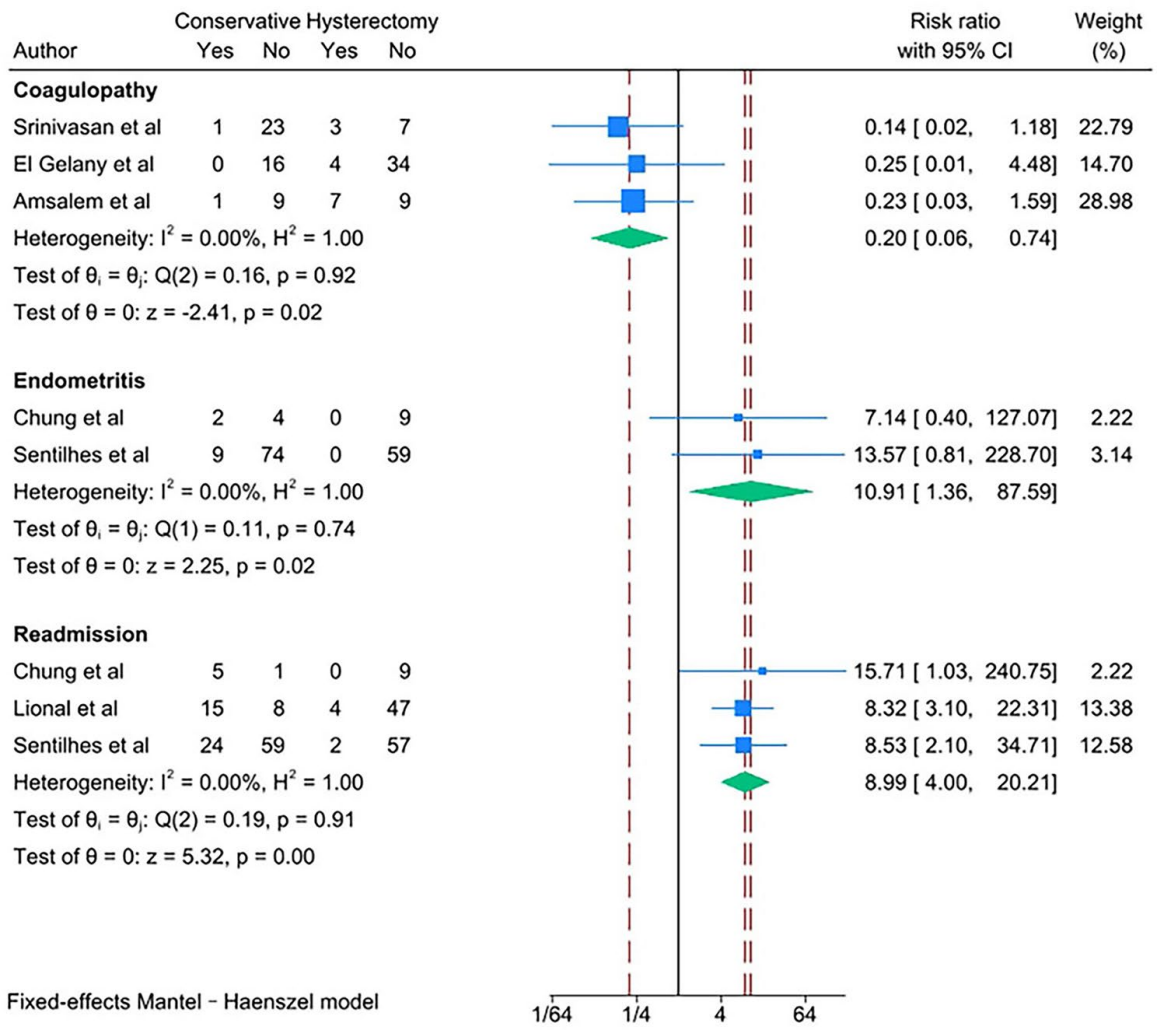
Coagulopathy, endometritis and readmission for PAS women undergoing conservative management or cesarean hysterectomy

**Fig. 7:**
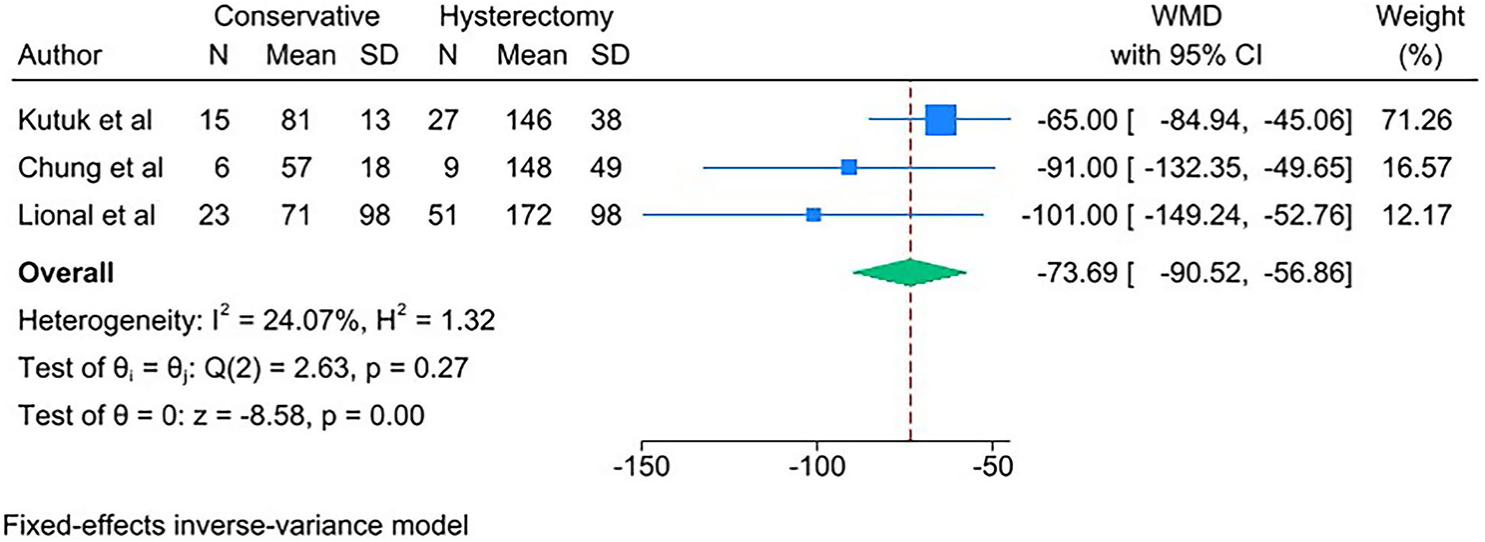
Mean operating time (min) for PAS women undergoing conservative management or cesarean hysterectomy

**Fig. 8:**
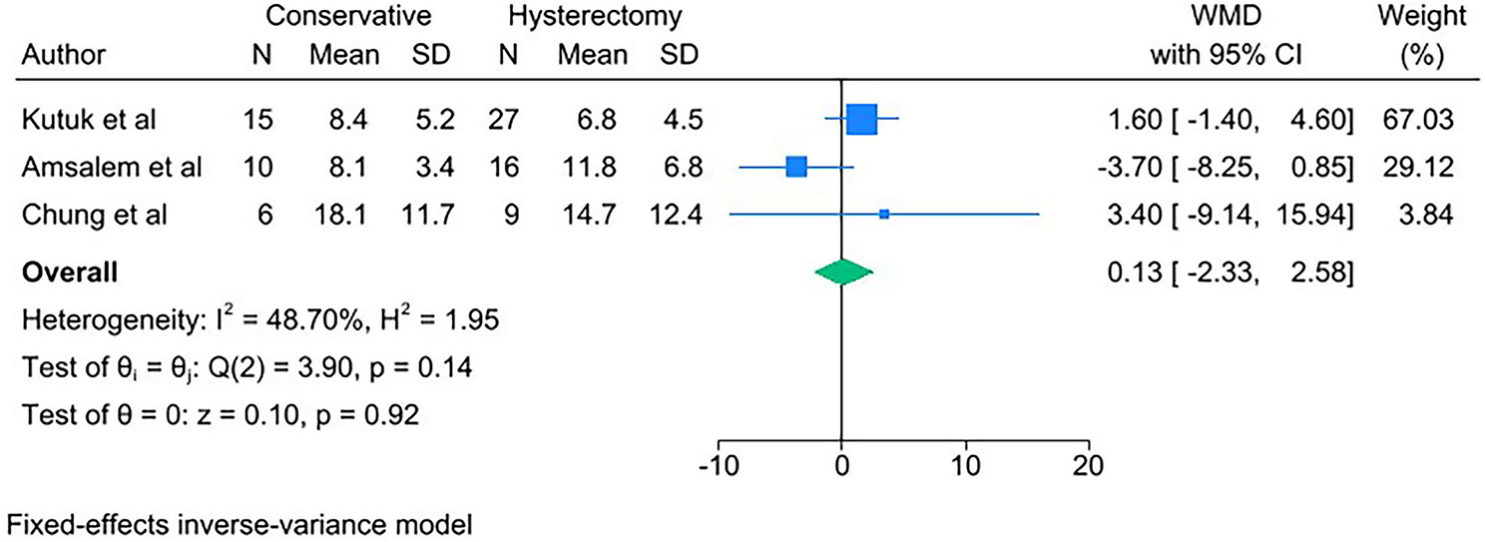
Hospital stays (days) for PAS women undergoing conservative management or cesarean hysterectomy

**Fig. 9:**
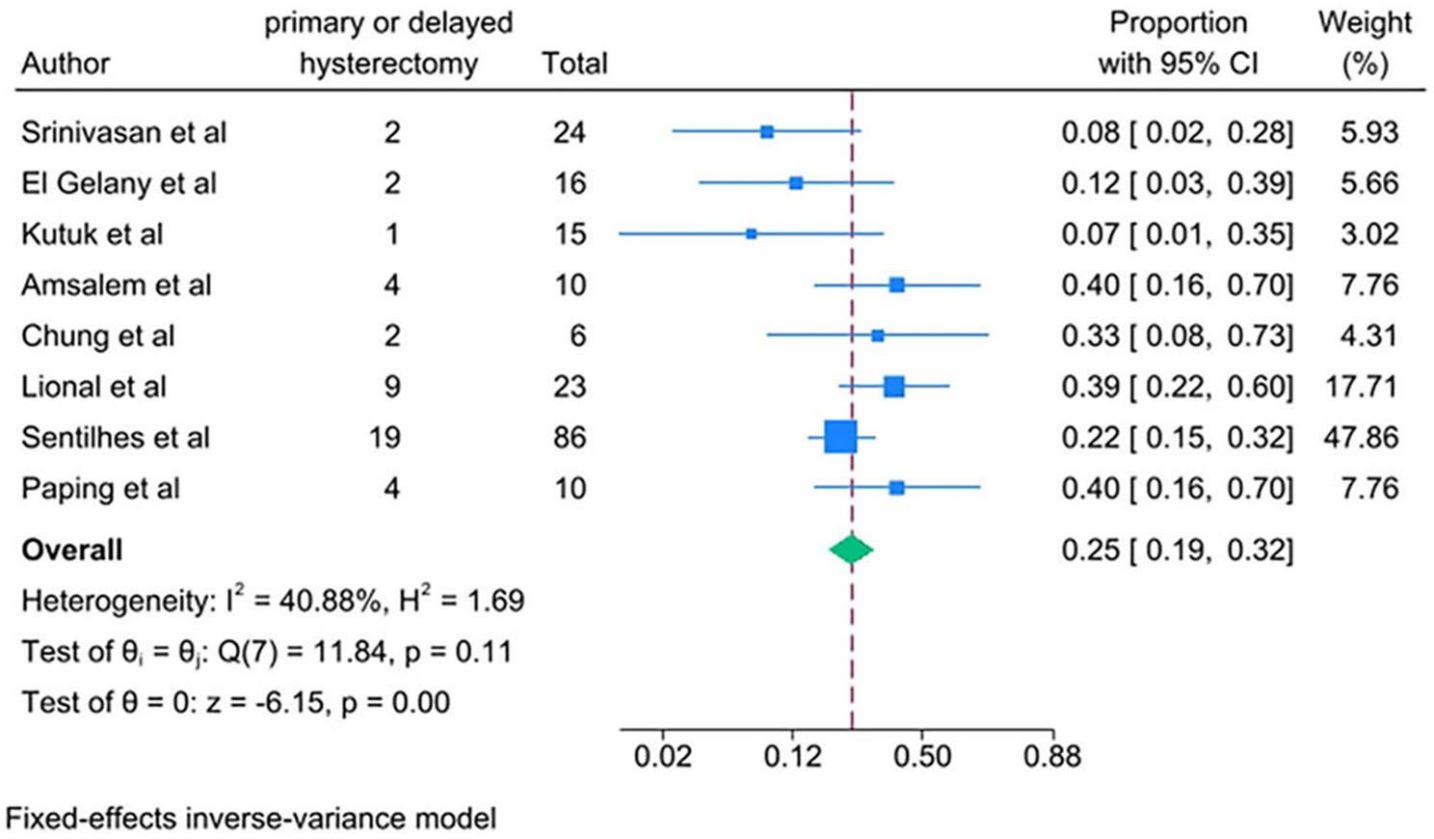
Hysterectomy incident for PAS women undergoing conservative management

**Fig. 10:**
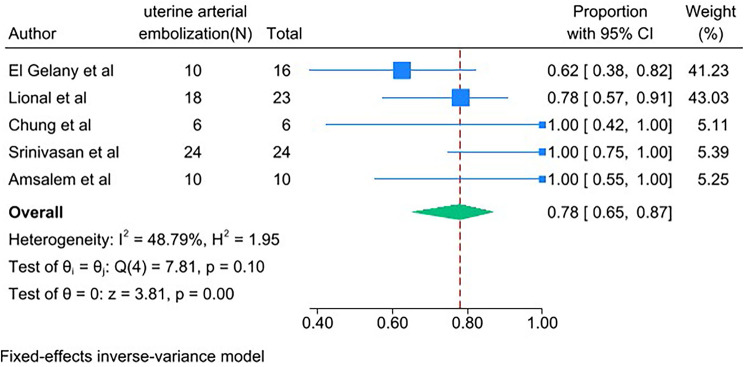
The use of uterine arterial embolization for PAS women undergoing conservative management

PAS women with conservative management decreased risks of the following outcomes compared with PAS women with cesarean hysterectomy: bladder injury [RR 0.24; 95% CI: 0.11, 0.50; *I*^*2*^ = 0.00%] (Fig. 4), ICU admission [RR 0.24; 95% CI: 0.11, 0.52; *I*^*2*^ = 0.00%] (Fig. 5) and coagulopathy [RR 0.20; 95% CI: 0.06, 0.74; *I*^*2*^ = 0.00%] (Fig. 6). Those with conservative management also had shorter mean operating time (in minutes) [WMD − 73.69; 95% CI: -90.52, -56.86; *I*^*2*^ = 24.07%] (Fig. 7). However, PAS women with conservative management increased risk for endometritis [RR 10.91; 95% CI: 1.36, 87.59; *I*^*2*^ = 0.00%] and readmission [RR 8.99; 95% CI: 4.00, 12.21; *I*^*2*^ = 0.00%] (Fig. 6). There was no statistically significant difference in hospital stays (in days) [WMD 0.13; 95% CI: -2.33, 2.58; *P>*0.1] (Fig. 8) between the two groups. The incidence of primary or delayed hysterectomy rate was 25% (95% CI: 19–32, *I*^*2*^ = 40.88%) (Fig. 9) and the use of uterine arterial embolization rate was 78% (95% CI: 65–87, *I*^*2*^ = 48.79%) (Fig. 10) in PAS women with conservative management.

#### Subgroup analysis

**Fig. 11:**
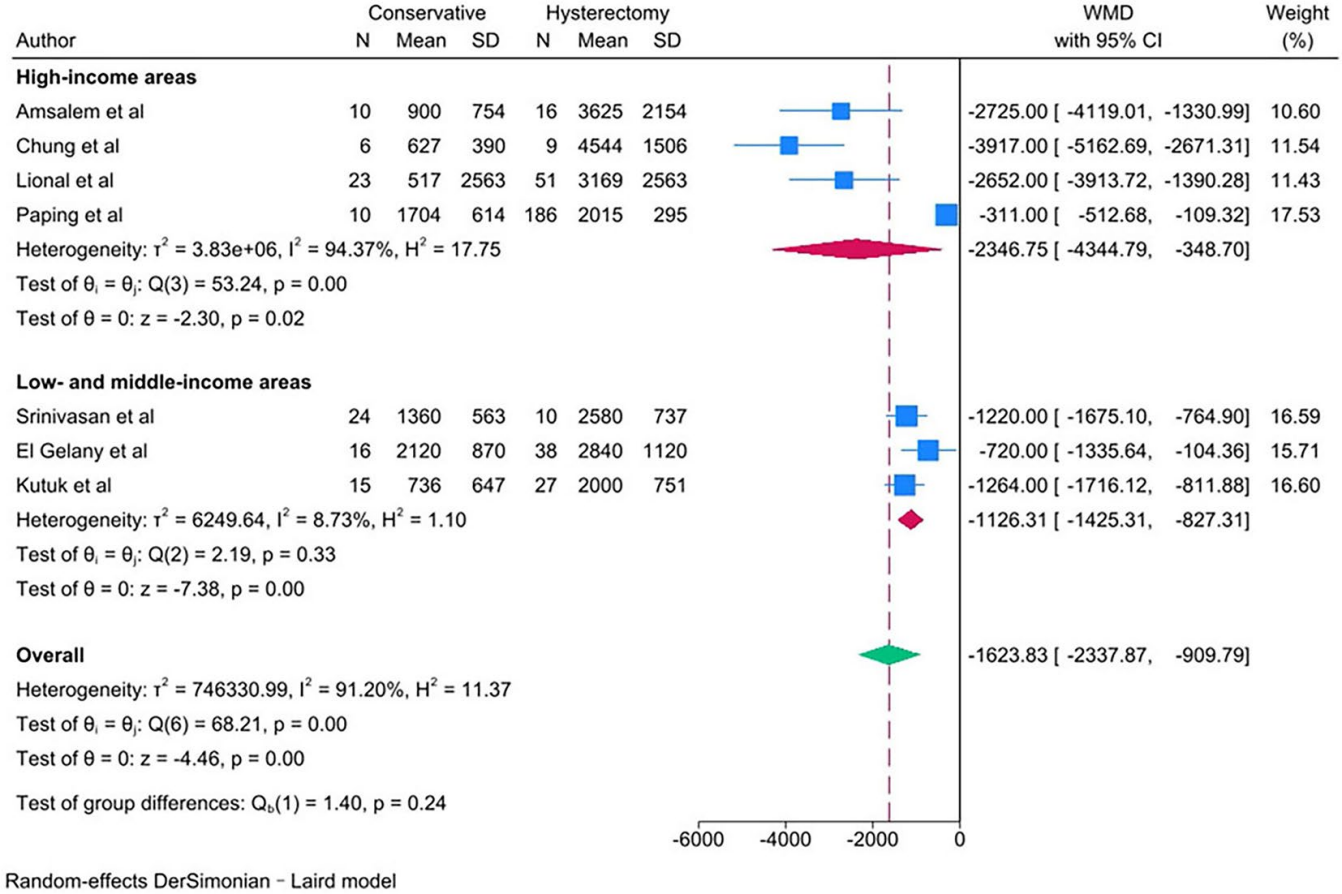
Subgroup analysis of estimated blood loss (mL) for PAS women undergoing conservative management or cesarean hysterectomy

**Fig. 12:**
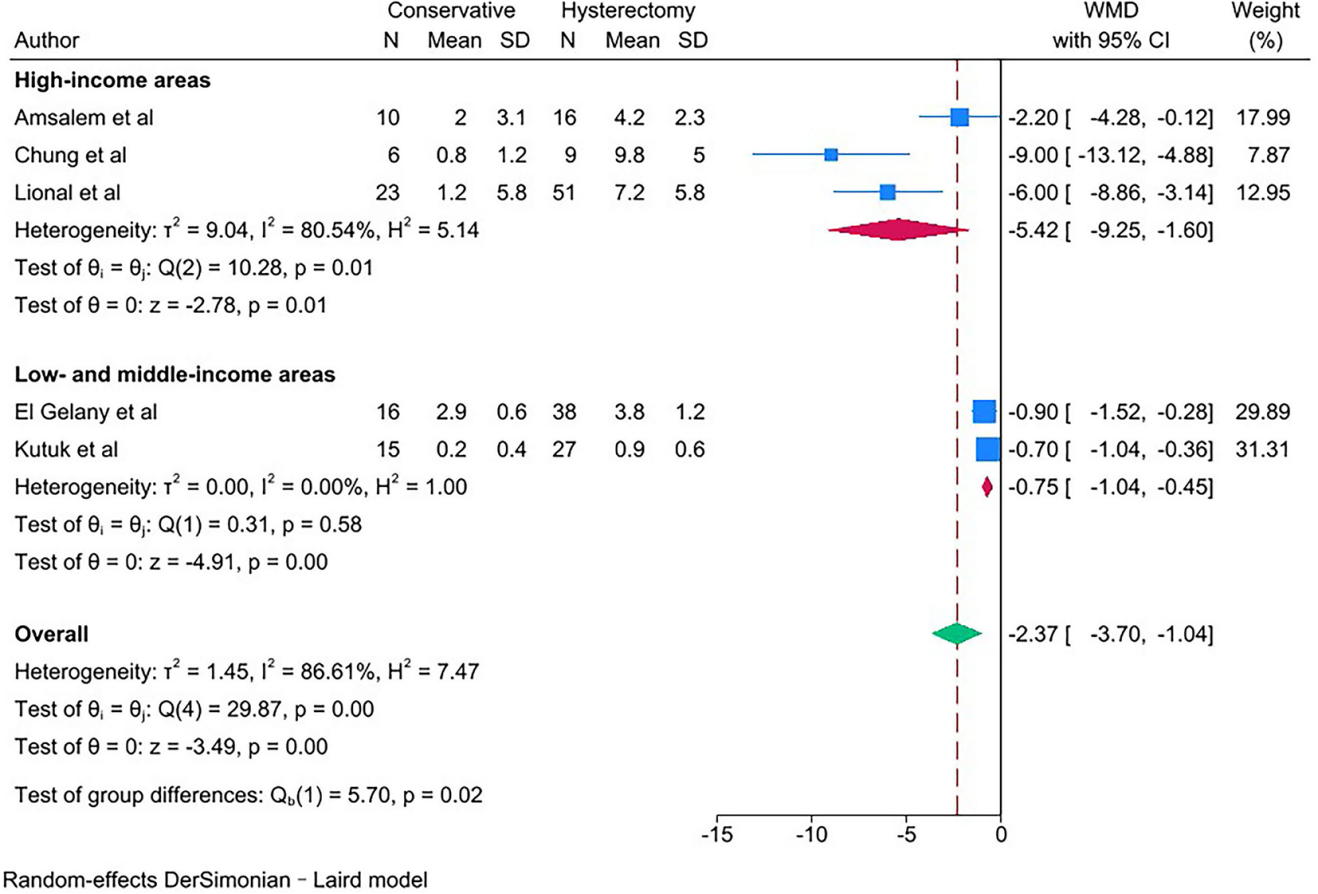
Subgroup analysis of packed red blood cells transfused (units) for PAS women undergoing conservative management or cesarean hysterectomy

The outcomes of estimated blood loss and units of packed red blood cells transfused were subgroup analyzed by area income because of the significant heterogeneity (*I*^*2*^ ≥ 50%). PAS women with conservative management showed observably lower estimated blood loss (in mL) [WMD − 2346.75; 95% CI: -4344.79, -348.70; *I*^*2*^ = 94.37%] (Fig. 11) and required observably fewer units of packed red blood cells transfused [WMD − 5.42; 95% CI: -9.25, -1.60; *I*^*2*^ = 80.54%] (Fig. 12) in high-income areas than PAS women with cesarean hysterectomy in high-income areas. But PAS women with conservative management showed not obviously lower estimated blood loss (in mL) [WMD − 1126.31; 95% CI: -1425.31, -827.31; *I*^*2*^ = 8.73%] (Fig. 11) and required not obviously fewer units of packed red blood cells transfused [WMD − 0.75; 95% CI: -1.04, -0.45; *I*^*2*^ = 0.00%] (Fig. 12) in low- and middle-income areas than PAS women with cesarean hysterectomy in low- and middle-income areas.

### Publication bias

Begg’s test did not indicate publication bias (*P* = 0.23 for estimated blood loss (in mL), *P* = 0.81 for the use of uterine arterial embolization). Egger’s test did not indicate publication bias (*P* = 0.79 for units of fresh frozen plasma transfused, *P* = 0.63 for bladder injury, *P* = 0.06 for ICU admission, *P* = 0.91 for coagulopathy, *P* = 0.10 for mean operating time (in minutes), *P* = 0.76 for endometritis, *P* = 0.68 for readmission, *P* = 0.76 for hospital stays (in days), *P* = 0.56 for primary or delayed hysterectomy). Although Egger’s and Begg’s tests did indicate publication bias for units of packed red blood cells transfused (*P* < 0.05), the main outcome did not change after using the Trim-and-fill method [WMD − 0.77; 95% CI: -2.16, -0.63].

## Discussion

### Principal findings and implications

The meta-analysis compared the maternal outcomes according to conservative management and cesarean hysterectomy for women with PAS. Those who had conservative management showed lower estimated blood loss, required fewer units of packed red blood cells transfused and units of fresh frozen plasma transfused, needed shorter mean operation time, and presented decreased risks of bladder injury, ICU admission and coagulopathy, but had an increased risks for endometritis and readmission, compared with women with PAS undergoing cesarean hysterectomy. There was no statistically significant difference in hospital stays between the two groups. Subgroup analysis showed that women with PAS treated conservativly had better effects on maternal outcomes of estimated blood loss and packed red blood cells transfusion in high-income areas than in low- and middle-income areas. The review also showed that the incidence of primary or delayed hysterectomy rate was 25% and the use of uterine arterial embolization rate was 78% for conservative management.

PAS disorders have become a momentous contributor to severe maternal morbidity. Predictors for PAS include previous cesarean delivery, placenta previa, assisted reproductive technologies (ART), uterine surgeries, multiparity, and advanced gestational age [25]. Due to the lack of randomized clinical trials, the best management strategy for pregnant women with PAS is still undefined worldwide [26]. A recent retrospective cohort study by Aryananda et al. showed that cesarean hysterectomy was significantly associated with higher mean blood loss (3168 ± 1916 mL vs. 1379 ± 769 mL), massive transfusion (35.3% vs. 2.5%), bladder injury (20.6% vs. 4.5%), DIC (5.9% vs. 0.5%) and ICU admission (32.4% vs. 1.5%) compared with uterine preservation in women with PAS [27], which were in line with our study. Our results were also in concordance with those of Nieto-Calvache et al. who included 75 women with PAS and indicated that cesarean delivery and hysterectomy group had a greater blood transfusion frequency (81.8% vs. 67.2%) and a longer operation time (216.5 min vs. 164.4 min) than the conservative group [28]. These findings demonstrated high postoperative morbidity of cesarean hysterectomy as a traditional therapeutic maneuver.

Currently, conservative management has been used in many institutions to treat women with PAS to minimize severe postoperative morbidity and preserve fertility. A recent study reviewing 17 women with PAS who were conservatively treated by leaving the placenta in situ indicated that the uterus retention rate was 88% and all women had favorable maternal outcomes [29]. Sentilhes et al. reviewed 167 women with PAS and showed that 78.4% of women avoided hysterectomy by conservative management [30]. The present study showed a 76% success rate for uterine preservation in conservative management by leaving the placenta in situ, which was similar to previously published findings. Nevertheless, conservative management was associated with higher rates of endometritis and readmission compared with cesarean hysterectomy, owing to the retention of the placenta in the uterine cavity [5], which was in line with our study. Some studies reported that subsequent fertility and pregnancy outcomes seemed unaffected after successful conservative management of PAS, and the main adverse pregnancy outcome in subsequent pregnancies was recurrence of PAS [20, 31]. Subgroup analyses showed that pregnant women with PAS who were treated conservatively in high-income areas had better outcomes in terms of estimated blood loss and transfusion of packed red blood cells compared to low- and middle-income areas, which tend to have adequate medical resources and specialized multidisciplinary teams. It follows that not all women with PAS are suitable for conservative management, as up to a third will experience prenatal or intraoperative bleeding [3]. The women with PAS may not be willing to accept the burden of long-term follow-up and the limitations of conservative management. FIGO recommended conservative management with specialized equipment and expert surgical teams, and leaving the placenta in situ is an option for women who agree to long-term follow-up in a professional medical center [32]. Conservative management may be a viable management strategy, but requires consideration of a variety of factors, including but not limited to individual disease characteristics, gestational age at delivery, surgical team experience, and institutional resources [33].

Selective transcatheter arterial embolization (TAE) is an effective treatment for uncontrolled postpartum hemorrhage, and the combination of TAE with cesarean delivery was used by many specialists in the conservative management of PAS [22, 23, 34]. Our study showed a 78% utilization rate of uterine arterial embolization in conservative management, including therapeutic or prophylactic embolization. A retrospective case-control study included 71 women diagnosed with PAS before cesarean delivery with or without prophylactic TAE and found that this technique could effectively reduce intraoperative hemorrhage and did not cause severe maternal outcomes [35]. A recent cohort study indicated that TAE was an effective alternative to hysterectomy for PPH, and subsequent fertility seemed to be limited in contrast to previous studies that concluded fertility was unaffected [36]. Large prospective follow-up studies are needed to confirm the long-term outcomes after the operation.

Overall, our study considered conservative management by leaving the placenta in situ to be an effective alternative to hysterectomy when women with PAS had a strong desire to preserve their uterus. Meanwhile, women with PAS who wish to be treated conservatively should be fully informed the advantages and limitations of the procedure by their obstetricians and radiologists. Furthermore, the decision to perform conservative management should consider logistic factors such as the differences of individual disease characteristics, the accessibility of adequate medical resources and the availability of multidisciplinary teams.

### Limitations and future research

The main strength of this meta-analysis was that it might be the first attempt to pool the results of existing studies comparing maternal outcomes in women with PAS who underwent conservative management and cesarean hysterectomy. This review was limited by the most literature included based on retrospective cohorts, and thus there might be a selection bias due to lack of adjustment for confounding factors. The statistical methods such as propensity score matching and the application of standardized definitions of disease staging could be used in future primary studies to reduce this limitation. Another limitation was that the specificity of women with PAS and the failure of data acquisition from the included study made the small number of included events, which might reduce the precision of the statistical results. Multi-center studies could be conducted to increase the sample size and make the findings more convincing. And there was a high degree of heterogeneity in some outcomes, which was addressed by using random effect model and conducting subgroup analyses. The reason for high heterogeneity could be the variable methods used by each institution to calculate indicators, such as total blood loss and units of blood transfused, as well as the different levels of medical care between developed and developing countries. In addition, there might be a regional bias because our paper included studies from only limited regions. More studies covering different regions need to be included to draw universally applicable conclusions.

## Conclusions

Women with PAS undergoing conservative management were associated with lower rates of blood loss, blood transfusion, hysterectomy, and major severe maternal morbidity than those of cesarean hysterectomy, but it was associated with higher rates of endometritis and readmission when compared with cesarean hysterectomy. Conservative management could be an effective alternative to hysterectomy when women with PAS desire to preserve the uterus and are informed about the limitations of conservative management.

### Electronic supplementary material

Below is the link to the electronic supplementary material.

Supplementary Table 1

Supplementary Table 2

Supplementary Figure 1

## Data Availability

All data are included in the tables.

## Abbreviations

PAS: Placenta accreta spectrum
DIC: Disseminated intravascular coagulation
ACOG: American College of Obstetricians and Gynecologists
FIGO: International Federation of Gynecology and Obstetrics
ICU: Intensive care unit
PRISMA: Systematic Reviews and Meta-Analyses
PROSPERO: International Prospective Register of Systematic Reviews
NOS: Scale Newcastle-Ottawa Quality Assessment Scale
RR: Risk ratio
95% CI: 95% confidence interval
WMD: Weighted mean difference
ART: Assisted reproductive technologies
TAE: Transcatheter arterial embolization
PPH: Postpartum hemorrhage

## References

[1] Hecht JL, Baergen R, Ernst LM, Katzman PJ, Jacques SM, Jauniaux E (2020). Classification and reporting guidelines for the pathology diagnosis of placenta accreta spectrum (PAS) disorders: recommendations from an expert panel. Mod Pathol.

[2] Silver RM, Branch DW (2018). Placenta Accreta Spectrum. N Engl J Med.

[3] Einerson BD, Gilner JB, Zuckerwise LC (2023). Placenta Accreta Spectrum. Obstet Gynecol.

[4] Bailit JL, Grobman WA, Rice MM, Reddy UM, Wapner RJ, Varner MW (2015). Morbidly adherent placenta treatments and outcomes. Obstet Gynecol.

[5] Sentilhes L, Seco A, Azria E, Beucher G, Bonnet MP, Branger B (2022). Conservative management or cesarean hysterectomy for placenta accreta spectrum: the PACCRETA prospective study. Am J Obstet Gynecol.

[6] Einerson BD, Silver RM, Jauniaux E (2021). Placenta accreta spectrum: welcome progress and a call for standardisation. BJOG.

[7] Silver RM (2016). Placenta accreta: we can do better!. BJOG.

[8] American College of O, Gynecologists, the Society for Maternal-Society of Gynecologic O, Fetal M, Cahill AG, Beigi R et al. Placenta Accreta Spectrum. Am J Obstet Gynecol. 2018;219(6):B2-B16. 10.1016/j.ajog.2018.09.042 30471891

[9] Chang WH, Chou FW, Wang PH (2023). The conservative management of pregnant women with placenta accreta spectrum remains challenging. Taiwan J Obstet Gynecol.

[10] Grover B, Einerson BD, Keenan KD, Gibbins KJ, Callaway E, Lopez S (2022). Patient-reported Health outcomes and Quality of Life after Peripartum Hysterectomy for Placenta Accreta Spectrum. Am J Perinatol.

[11] Wang Q, Ma J, Zhang H, Dou R, Huang B, Wang X (2022). Conservative management versus cesarean hysterectomy in patients with placenta increta or percreta. J Matern Fetal Neonatal Med.

[12] Jauniaux E, Ayres-de-Campos D, Diagnosis FPA (2018). Management Expert Consensus P. FIGO consensus guidelines on placenta accreta spectrum disorders: introduction. Int J Gynaecol Obstet.

[13] Gallucci E, Tucci C, Zullino S, Ottanelli S, Rambaldi MP, Bruscoli G (2023). Follow up of PAS (placenta accreta spectrum) disorders treated with conservative management. Ital J Gynaecol Obstet.

[14] Herzberg S, Ezra Y, Haj Yahya R, Weiniger CF, Hochler H, Kabiri D. Long-term gynecological complications after conservative treatment of placenta accreta spectrum. Front Med. 2022;9. 10.3389/fmed.2022.992215 PMC9650034 36388950

[15] Page MJ, McKenzie JE, Bossuyt PM, Boutron I, Hoffmann TC, Mulrow CD (2021). The PRISMA 2020 statement: an updated guideline for reporting systematic reviews. BMJ.

[16] Hootman JM, Driban JB, Sitler MR, Harris KP, Cattano NM (2011). Reliability and validity of three quality rating instruments for systematic reviews of observational studies. Res Synth Methods.

[17] Hunter JP, Saratzis A, Sutton AJ, Boucher RH, Sayers RD, Bown MJ (2014). In meta-analyses of proportion studies, funnel plots were found to be an inaccurate method of assessing publication bias. J Clin Epidemiol.

[18] Amsalem H, Kingdom JCP, Farine D, Allen L, Yinon Y, D’Souza DL (2011). Planned caesarean hysterectomy Versus conserving caesarean section in patients with Placenta Accreta. J Obstet Gynaecol Can.

[19] Chung MY, Cheng YK, Yu SC, Sahota DS, Leung TY (2013). Nonremoval of an abnormally invasive placenta at cesarean section with postoperative uterine artery embolization. Acta Obstet Gynecol Scand.

[20] El Gelany S, Mosbeh MH, Ibrahim EM, Mohammed M, Khalifa EM, Abdelhakium AK (2019). Placenta Accreta Spectrum (PAS) disorders: incidence, risk factors and outcomes of different management strategies in a tertiary referral hospital in Minia, Egypt: a prospective study. BMC Pregnancy Childbirth.

[21] Kutuk MS, Ak M, Ozgun MT (2018). Leaving the placenta in situ versus conservative and radical surgery in the treatment of placenta accreta spectrum disorders. Int J Gynaecol Obstet.

[22] Lional KM, Tagore S, Wright AM (2020). Uterine conservation in placenta accrete spectrum (PAS) disorders: a retrospective case series: is expectant management beneficial in reducing maternal morbidity?. Eur J Obstet Gynecol Reprod Biol.

[23] Srinivasan B, Rani U, Palaniappan N, Vijayaraghavan J, Vishwanath U, Lakshmi V (2021). Study on outcomes of pregnancy in women with Placenta Accreta Spectrum: a 10-year study in a Tertiary Care Center. J South Asian Feder Obs Gynae.

[24] Paping A, Bluth A, Al Naimi A, Mhallem M, Kolak M, Jaworowski A et al. Opportunities for, and barriers to, uterus-preserving surgical techniques for placenta accreta spectrum. Acta Obstet Gynecol Scand. 2024. 10.1111/aogs.14855 PMC12087390 38695676

[25] Carusi DA (2018). The Placenta Accreta Spectrum: epidemiology and risk factors. Clin Obstet Gynecol.

[26] Zhong W, Zhu F, Li S, Chen J, He F, Xin J (2021). Maternal and neonatal outcomes after Planned or Emergency Delivery for Placenta Accreta Spectrum: a systematic review and Meta-analysis. Front Med (Lausanne).

[27] Aryananda RA, Aditiawarman A, Gumilar KE, Wardhana MP, Akbar MIA, Cininta N (2022). Uterine conservative-resective surgery for selected placenta accreta spectrum cases: Surgical-vascular control methods. Acta Obstet Gynecol Scand.

[28] Nieto-Calvache AJ, Palacios-Jaraquemada JM, Aryananda R, Basanta N, Aguilera R, Benavides JP (2023). How to perform the one-step conservative surgery for placenta accreta spectrum move by move. Am J Obstet Gynecol MFM.

[29] Wong YF, Lo TK, Chan VYT, Ng VKS, Yung WK, Tsang HH et al. Conservative management for placenta accreta spectrum disorders: experience of a regional hospital from 2013 to 2021. Hong Kong J Gynaecol Obstet Midwifery. 2023;23(2).

[30] Sentilhes L, Ambroselli C, Kayem G, Provansal M, Fernandez H, Perrotin F (2010). Maternal outcome after conservative treatment of placenta accreta. Obstet Gynecol.

[31] Sentilhes L, Kayem G, Ambroselli C, Provansal M, Fernandez H, Perrotin F (2010). Fertility and pregnancy outcomes following conservative treatment for placenta accreta. Hum Reprod.

[32] Sentilhes L, Kayem G, Chandraharan E, Palacios-Jaraquemada J, Jauniaux E, Diagnosis FPA (2018). FIGO consensus guidelines on placenta accreta spectrum disorders: conservative management. Int J Gynaecol Obstet.

[33] Pineles BL, Sibai BM, Sentilhes L (2023). Is conservative management of placenta accreta spectrum disorders practical in the United States?. Am J Obstet Gynecol MFM.

[34] Sebastian B, Rajesh U, Scott PM, Sayeed S, Robinson GJ, Ettles DF (2023). Prophylactic uterine artery embolization in Placenta Accreta Spectrum-An active intervention to reduce morbidity and promote uterine preservation. J Vasc Interv Radiol.

[35] Xie L, Wang Y, Luo FY, Man YC, Zhao XL (2017). Prophylactic use of an infrarenal abdominal aorta balloon catheter in pregnancies complicated by placenta accreta. J Obstet Gynaecol.

[36] Radan AP, Schneider S, Zdanowicz JA, Raio L, Mertineit N, Heverhagen JT et al. Obstetrical and fertility outcomes following transcatheter pelvic arterial embolization for Postpartum Hemorrhage: a Cohort Follow-Up study. Life (Basel). 2022;12(6). 10.3390/life12060892 PMC9228119 35743923

