# Supplementary material for: Maternal outcomes of conservative management and cesarean hysterectomy for placenta accreta spectrum disorders: a systematic review and meta-analysis
Source: BMC Pregnancy Childbirth. 2024 Jul 5;24:463. doi: 10.1186/s12884-024-06658-x (PMC11227152; doi:10.1186/s12884-024-06658-x)
Supplement: Supplementary file 1 — Supplementary Table 1 [file 12884_2024_6658_MOESM1_ESM.docx]

**Table S1 The detailed search strategies for each database.**

| **Search strategy** | **Medline** |
| --- | --- |
| #1 "Placenta Accreta"[Mesh]  #2 (Placenta accreta[Title/Abstract]) OR (placenta increta[Title/Abstract]) OR (placenta percreta[Title/Abstract]) OR (abnormally invasive placenta[Title/Abstract]) OR (morbidly adherent placenta[Title/Abstract]) OR (placental anomalies[Title/Abstract])  #3 #1 OR #2  #4 Conservative treatment[Mesh]  #5 (Conservative delivery[Title/Abstract]) OR (Conservative management*[Title/Abstract]) OR (Conservative treatment*[Title/Abstract]) OR (Conservative therapy[Title/Abstract]) OR (Conservative therapies[Title/Abstract]) OR (uterine preservation[Title/Abstract])  #6 #4 OR #5  #7 #3 AND #6 | |

| **Search strategy** | **Embase** |
| --- | --- |
| #1. 'placenta accreta'/exp  #2. placenta AND increta:ti,ab  #3. placenta AND percreta:ti,ab  #4. abnormally AND invasive AND placenta:ti,ab  #5. morbidly AND adherent AND placenta:ti,ab  #6. placental AND anomalies:ti,ab  #7. #1 OR #2 OR #3 OR #4 OR #5 OR #6  #8. 'conservative treatment'/exp OR 'conservative treatment'  #9. conservative AND delivery:ti,ab  #10. conservative AND management*:ti,ab  #11. conservative AND treatment*:ti,ab  #12. conservative AND therapy:ti,ab  #13. conservative AND therapies:ti,ab  #14. 'uterine preservation':ti,ab  #15. #8 OR #9 OR #10 OR #11 OR #12 OR #13 OR #14 | |

| **Search strategy** | **Cochrane** |
| --- | --- |
| #1 MeSH descriptor: [Placenta Accreta] explode all trees  #2 (Placenta accreta):ti,ab,kw OR (placenta increta):ti,ab,kw OR (placenta percreta):ti,ab,kw OR (abnormally invasive placenta):ti,ab,kw OR (morbidly adherent placenta):ti,ab,kw  #3 (placental anomalies):ti,ab,kw  #4 #2 OR #  #5 #1 OR #  #6 MeSH descriptor: [Conservative Treatment] explode all trees  #7 (Conservative delivery):ti,ab,kw OR (Conservative management*):ti,ab,kw OR (Conservative treatment*):ti,ab,kw OR (Conservative therapy):ti,ab,kw OR (Conservative therapies):ti,ab,kw  #8 (uterine preservation):ti,ab,kw  #9 #7 OR #8  #10 #6 OR #9  #11 #5 and #10 | |

| **Search strategy** | **Web of Science** |
| --- | --- |
| 1:((((TS= (Placenta accreta) OR TS= (placenta increta)) OR TS=(placenta percreta)) OR TS=(abnormally invasive placenta)) OR TS=(morbidly adherent placenta)) OR TS=(placental anomalies)  2:(((((TS=(Conservative delivery)) OR TS=(Conservative management*)) OR TS=(Conservative treatment*)) OR TS=(Conservative therapy)) OR TS=(Conservative therapies)) OR TS=(uterine preservation)  3: #1 AND #2 | |

| **Search strategy** | **Sinomed** |
| --- | --- |
| 1. "placenta percreta" 2. "placenta implantation" OR "placenta increta" OR "Placenta accreta" OR "placenta percreta" 3. (#2) OR (#1) 4. "conservative treatment" 5. "conservative treatment" OR "conservative management" OR "conservative therapy" OR "uterine preservation" 6. (#5) OR (#4) 7. (#6) AND (#3) | |

| **Search strategy** | **CNKI** |
| --- | --- |
| 1: title: placenta percreta OR (full text or abstract or key words: placenta percreta + placenta implantation + placenta increta + placenta accreta  2: title: conservative treatment OR (full text or abstract or key words: conservative treatment + conservative management + conservative therapy + uterine preservation)  3: #1 AND #2 | |

| **Search strategy** | **Chinese Wangfang database** |
| --- | --- |
| 1: title: (conservative treatment OR conservative management OR conservative therapy OR uterine preservation)  2: title: (placenta increta OR placenta percreta OR placenta implantation OR placenta accreta)  3: #1 AND #2 | |

| **Search strategy** | **Chinese VIP database** |
| --- | --- |
| 1: Title/Abstract = conservative treatment OR Title/Abstract = conservative management OR Title/Abstract = conservative therapy OR Title/Abstract = uterine preservation  2: Title/Abstract = placenta increta OR Title/Abstract = placenta implantation) OR Title/Abstract = placenta accreta OR Title/Abstract = placenta implantation OR Title/Abstract = placenta percreta  3: #1 AND #2 | |
