# Supplementary material for: Maternal outcomes of conservative management and cesarean hysterectomy for placenta accreta spectrum disorders: a systematic review and meta-analysis
Source: BMC Pregnancy Childbirth. 2024 Jul 5;24:463. doi: 10.1186/s12884-024-06658-x (PMC11227152; doi:10.1186/s12884-024-06658-x)
Supplement: Supplementary file 2 — Supplementary Table 2 [file 12884_2024_6658_MOESM2_ESM.docx]

| **The Newcastle-Ottawa Scale(NOS) quality assessment of the included studies in this meta-analysis** | | | | | | | | | |
| --- | --- | --- | --- | --- | --- | --- | --- | --- | --- |
| **Cohort Star Template** | | | | | | | | | |
| **Study** | **Selection of cohort** | | | | **Comparability of cohorts** | **Outcome** | | | **Total(9)** |
|  | Representativeness of the exposed cohort | Selection of the non exposed cohort | Ascertainment of exposure | Demonstration that outcome of interest was not present at start of study | Comparability of cohorts on the basis of the design or analysis | Assessment of outcome | Was follow-up long enough for outcomes to occur | Adequacy of follow up of cohorts |  |
| **Srinivasan, B.** | ☆ | ☆ | ☆ | ☆ |  | ☆ | ☆ | ☆ | 7 |
| **El Gelany, S.** | ☆ | ☆ | ☆ | ☆ | ☆ | ☆ |  | ☆ | 7 |
| **Kutuk, M.S.** | ☆ | ☆ | ☆ | ☆ | ☆ | ☆ | ☆ | ☆ | 8 |
| **Amsalem, H.** | ☆ | ☆ | ☆ | ☆ | ☆ | ☆ | ☆ | ☆ | 8 |
| **Chung, M.Y.** | ☆ | ☆ | ☆ | ☆ | ☆ | ☆ | ☆ | ☆ | 8 |
| **Lional, K.M.** | ☆ | ☆ | ☆ | ☆ |  | ☆ | ☆ | ☆ | 7 |
| **Sentilhes, L.** | ☆ | ☆ | ☆ | ☆ | ☆☆ | ☆ | ☆ | ☆ | 9 |
| **Paping, A.** | ☆ | ☆ | ☆ | ☆ | ☆ | ☆ | ☆ | ☆ | 8 |

**Table S2 Summary of risk of bias in the seven studies included in the review**

☆, 1 point; ☆☆, 2 points
