# Supplementary figures and images for: Maternal outcomes of conservative management and cesarean hysterectomy for placenta accreta spectrum disorders: a systematic review and meta-analysis
Source: BMC Pregnancy Childbirth. 2024 Jul 5;24:463. doi: 10.1186/s12884-024-06658-x (PMC11227152; doi:10.1186/s12884-024-06658-x)

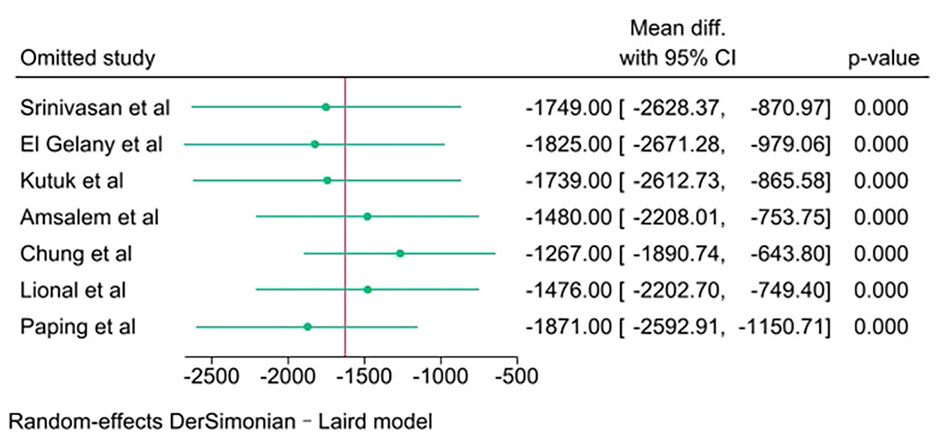

Supplement: Supplementary file 3 — Supplementary Figure 1 [file 12884_2024_6658_MOESM3_ESM.jpg]
